# Effects of malleable kinetochore morphology on measurements of intrakinetochore tension

**DOI:** 10.1098/rsob.200101

**Published:** 2020-07-08

**Authors:** Fioranna Renda, Valentin Magidson, Irina Tikhonenko, Rebecca Fisher, Christopher Miles, Alex Mogilner, Alexey Khodjakov

**Affiliations:** 1 New York State Department of Health, Wadsworth Center, Albany, NY, USA; 2 Courant Institute and Department of Biology, New York University, New York, NY, USA; 3 Rensselaer Polytechnic Institute, Troy, NY, USA

**Keywords:** mitosis, kinetochores, intrakinetochore tension, Taxol, mitotic spindle

## Abstract

The distance between fluorescent spots formed by various kinetochore proteins (delta) is commonly interpreted as a manifestation of intrakinetochore tension (IKT) caused by microtubule-mediated forces. However, large-scale changes of the kinetochore architecture (such as its shape or dimensions) may also contribute to the value of delta. To assess contributions of these non-elastic changes, we compare behaviour of delta values in human kinetochores with small yet mechanically malleable kinetochores against compound kinetochores in Indian muntjac (IM) cells whose architecture remains constant. Due to the micrometre-scale length of kinetochore plates in IM, their shape and orientation are discernible in conventional light microscopy, which enables precise measurements of IKT independent of contributions from changes in overall architecture of the organelle. We find that delta in IM kinetochores remains relatively constant when microtubule-mediated forces are suppressed by Taxol, but it prominently decreases upon detachment of microtubules. By contrast, large decreases of delta observed in Taxol-treated human cells coincide with prominent changes in length and curvature of the kinetochore plate. These observations, supported by computational modelling, suggest that at least 50% of the decrease in delta in human cells reflects malleable reorganization of kinetochore architecture rather than elastic recoil due to IKT.

## 1. Introduction

Segregation of chromosomes during cell division (mitosis) depends upon kinetochores, macromolecular assemblies located at the centromere of each chromosome. Kinetochores perform two principal functions: they generate the force that propels chromosomes and produce a checkpoint signal that delays progression through mitosis until all chromosomes attach to spindle microtubules. Kinetochores comprise over a hundred of various proteins [1]. Furthermore, as the cell progresses through mitosis, molecular composition of the kinetochore and its architecture change in response to various types of interactions with spindle microtubules [2,3]. These adaptive changes in size and shape of the kinetochores ensure that microtubule attachments form rapidly yet with a low number of errors [4,5]. Thus, revealing mechanisms that govern kinetochore architecture is of significant interest.

Due to its small size in most mammalian cells (approx. 300 nm), the shape of the kinetochore or the distribution of its components cannot be directly delineated in conventional light microscopy (LM). A popular approach to overcoming this limitation is based on measuring the ‘delta', a distance between centroids of fluorescent spots formed by various proteins, visualized in different colours within the same kinetochore. Measurements of delta lay the foundation of a nanometre-scale map that attributes various proteins to thin layers orthogonal to the inner–outer (from the centromere towards attached microtubules) axis of the kinetochore [6,7]. Delta between the proteins at the base of the kinetochore (e.g. CenpA) and those in the microtubule-binding domain (e.g. Hec1) has been shown to decrease when microtubule dynamics are suppressed by Taxol [7,8]. The decrease was interpreted as a manifestation of changes in the physical separation between the inner and outer layers, which in turn led to the concept of ‘intra-kinetochore tension' and the notion that stretching of the kinetochore is necessary for the satisfaction of the spindle assembly checkpoint (SAC) [9]. This attractive hypothesis, however, remains debatable for two main reasons. First, there exists a high degree of variability in delta measurements conducted via different techniques and in different laboratories. Some report an approximately 30 nm (22–27%) decrease in delta between CenpA-GFP and Hec1 in human cells treated with Taxol [7,10], while others observe no statistically significant change in separation of the same kinetochore proteins under similar experimental conditions [11,12]. These discrepancies probably arise from the alternative measuring techniques, particularly various approaches to compensating chromatic aberration, inevitable in LM [6,7,10,12,13]. Second, interpretation of delta as a metric for physical distances between molecules is obfuscated by the poor characterization of larger-scale architecture of the kinetochore, such as the shape and dimensions of these organelles upon various physiological conditions. In single-molecule high-resolution colocalization (SHREC) [14,15] analysis of negligibly small shapeless light sources, the value of delta depends only on the positions of molecules. For larger objects, the distance between the centres of mass for different components reflects both the average separation of these components and the shape of the object. Applicability of SHREC to the kinetochore (termed kSHREC [7]) was justified by the layered-disc kinetochore morphology in electron microscopy (EM) [16], which was assumed to remain constant. More recent observations of significant alterations in the size and shape of the kinetochore in response to various types of interactions with microtubules [4,5,17,18] challenge this assumption [10]. The ongoing debate on the importance of intrakinetochore tension (IKT) prompted us to evaluate contributions of changes in distances between kinetochore layers versus changes in shape and dimensions of the kinetochores towards the value of delta. To this end, we applied kSHREC analysis to the compound kinetochores of Indian muntjac (IM) formed via lateral fusion of centromeres during evolution of this species. IM kinetochores typically comprise a trilaminar plate of roughly 75 nm thickness; however, the length of the plate exceeds 1.5 µm, compared with approximately 0.3 µm for human cells. This morphology makes the shape and orientation of the kinetochore discernible in LM. Comparative analyses suggest that changes in the length and curvature of the kinetochore plate induced by Taxol in human but not in IM cells contribute heavily to the decrease in delta. Furthermore, our data suggest a large degree of spatial intermix among the inner- and outer-kinetochore components. These features of kinetochore architecture need to be considered in interpretation of kSHREC and the role of IKT in the control of mitotic progression.

## 2. Results

### 2.1. Advantages of Indian muntjac kinetochores for kSHREC analysis

**Figure 1.**
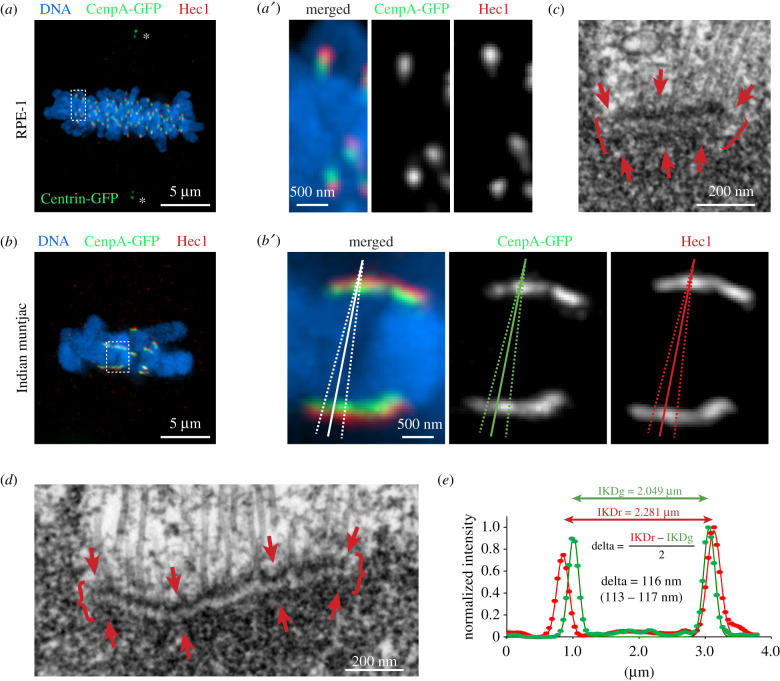
Kinetochore architecture in human and Indian muntjac cells and the approach to delta measurements. (*a*) Human (RPE1) cell with inner and outer kinetochore domains labelled via expression of CenpA-GFP (green) and immunostaining for Ndc80/Hec1 (red). Paraformaldehyde (PFA) fixation. DNA is counterstained with Hoechst 33342 (blue). Maximum-intensity projection through the entire cell. Centrioles (asterisks) are labelled via expression of Centrin-1-GFP. (*a*′) Higher magnification of sister kinetochores boxed in *a*. Both inner (CenpA) and outer (Hec1) kinetochore proteins appear as small spots. (*b*) Indian muntjac (IM) cell with inner and outer kinetochore domains labelled as in *a*. PFA fixation. (*b*′) Higher magnification of sister kinetochores boxed in *b*. Inner and outer kinetochore proteins form thin layers with discernible orientation. Solid lines denote positions of scan profiles presented in *e*. Dashed lines mark the cone where multiple scans were performed to test variability of delta values. (*c*) A 70 nm EM section through a kinetochore in metaphase RPE1 cell. Arrows denote the trilaminar plate comprising approximately 25 nm thin electron-dense inner and outer layers (arrows) separated by approximately 25 nm thin translucent middle layer. The plate is approximately 300 nm long (curly brackets). (*d*) A 70 nm EM section through a kinetochore in IM metaphase. Arrows and curly brackets as in *c*. The plate is approximately 1000-nm long and approximately 75 nm wide. (*e*) Line profiles across co-planar sister kinetochores in IM scanned corresponding to the solid lines in *b*'. Markers are pixel intensities; lines are Gaussian fits. Inter-kinetochore distances IKDg and IKDr are the distances between maxima of the two green and two red peaks correspondingly. Delta is determined as half of the difference between IKDr and IKDg. Range of delta values is shown for multiple scans within the cone denoted by dashed lines in *b*′.

The low number of chromosomes in Indian muntjac (IM) arose from the fusion of numerous normally sized chromosomes during evolution of this species [19]. As a result, each IM kinetochore comprises tandem repeats of the conventional mammalian kinetochores [20]. While typical mammalian kinetochores appear as spots in conventional fluorescence LM (figure 1*a,a*′), IM kinetochores form thin lines with length greater than 1 µm (figure 1*b*,*b′*). The molecular composition of IM kinetochores is similar to that of conventional kinetochores [21], and major kinetochore components can be visualized in both via expression of fluorescently labelled proteins or antibody staining with similar efficiency (figure 1*a,b*). At the EM level, IM kinetochores display the typical trilaminar plate with the widths of the layers similar to those observed in other mammalian cells [16,22,23]. However, IM plates are several times longer than the 250–300 nm plates observed in human cells (figure 1*c,d*).

Because orientation of sister kinetochores is discernible in IM, intensity profiles can be generated by scanning a line approximately orthogonal to both plates (figure 1*b*′). Fitting these profiles with Gaussian functions determines positions of the fluorescent peaks with nanometre precision (figure 1*e*), and the distance between the green and red peaks within a kinetochore (i.e. delta) is then calculated as shown in figure 1*e*. Pairwise calculation of Delta for sister kinetochores negates chromatic aberration [7], and the accuracy of this approach is not affected by large standard deviations in the distribution of experimental values [10], which has been shown to erroneously increase mean values when delta is measured independently for each kinetochore [6,10]. A major limitation of the pairwise delta measurement in human cells is that this approach underestimates the value of delta if sister kinetochores swivel around the centromere so that their outer layers do not face in opposite directions [7,10]. However, because the plates of IM kinetochores are discernible in LM, this limitation is overcome by selecting approximately antiparallel sister kinetochores. Importantly, due to nonlinearity of trigonometric functions even relatively large deviations from orthogonality between the scan profile and long kinetochore plates result in relatively minor errors. Indeed, we find that delta fluctuates by less than 5% when scan lines are intentionally skewed by approximately 10° (figure 1*b*′,*e*). This behaviour is expected, as a 10° slant introduces a mere approximately 1.5% error [cos(20°) = 0.9848] while a visually intolerable 20° deviation from orthogonality would increase the error to just approximately 6%. By contrast, approximately 20° swivelling of spot-like human kinetochores leads to approximately 20% underestimation of delta in 1D measurements ('Method I' in [10]). Thus, delta measured via line-scans in IM achieves a precision not possible in kSHREC analyses of smaller human kinetochores whose orientation cannot be accurately determined.

### 2.2. Delta (CenpA-Hec1) decreases prominently in the absence of microtubules but minimally upon suppression of microtubule-mediated forces

**Figure 2.**
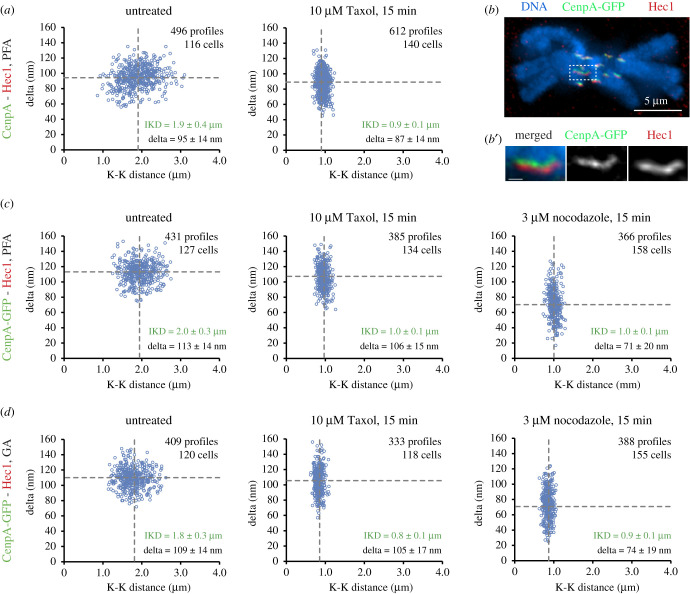
Delta does not decrease significantly in Taxol-treated IM cells. (*a*) Scatterplots of CenpA (green)–Hec1 (red) delta versus interkinetochore (CenpA–CenpA) distance for untreated metaphase and Taxol-treated IM cells. PFA fixation, followed by methanol. Dashed lines denote mean values. Mean ± s.d. also shown numerically. (*b*) IM cell with inner and outer kinetochore domains labelled via expression of CenpA-GFP (green) and immunostaining for Ndc80/Hec1 (red). Glutaraldehyde (GA) fixation. Note that the intensity of staining is similar to figure 1*b* although chromosome arms appear slightly more condensed. (*b*′) Higher magnification of sister kinetochores boxed in *b*. Scale bar = 500 nm. (*c*) Scatterplots of CenpA-GFP (green)–Hec1 (red) delta versus interkinetochore (CenpA-GFP–CenpA-GFP) distance for untreated, Taxol- and nocodazole-treated IM cells. PFA fixation. Mean ± s.d. shown. (*d*) As in *c* but cells are fixed with GA.

We focus our analyses on the separation between the microtubule-binding protein Hec1 and the centromere-specific histone CenpA that forms the base of the kinetochore. This pair of proteins displays the largest change of delta in Taxol-treated human cells [7,10]. To facilitate direct comparison of the results, the antibodies against Hec1 (9G3, Abcam) and CenpA (3–19, Abcam) are the same as in previous delta measurements in human cells [6,7,10]. We find that delta_CenpA−Hec1_ in untreated IM metaphase (95 ± 14 nm; figure 2*a*) is similar to that measured in human cells (approx. 90 nm [6,10]). Brief exposure to Taxol decreases delta_CenpA−Hec1_ to 87 ± 14 nm in IM (figure 2*a*). This decrease is statistically significant (*p* = 0.0006 in two-tailed Student's *t*-test); however, it is considerably smaller than the approximately 30 nm decrease observed in human cells [6,10,24].

Immunolocalization of endogenous CenpA requires partial denaturation of the sample because this protein, shielded by the DNA, is not accessible to the antibodies when chromatin structure is intact. Consequently, under conditions required for immunolocalization of CenpA, neither microtubules nor the trilaminar appearance of kinetochore plates are preserved as evidenced in EM [25,26]. To minimize potential effects of sample denaturation we analyse delta in IM cells that constitutively express CenpA-GFP. Fluorescence of this protein is preserved when cells are fixed with 3.2% PFA (figure 1*b*) or 1% GA (figure 2*b*,*b′*), which is important as fluorescent spots containing outer-kinetochore proteins are enlarged in human cells fixed with paraformaldehyde (PFA) versus glutaraldehyde (GA) [10]. This difference indicates differences in preservation of kinetochore morphology after GA versus PFA fixation.

We find that delta_CenpA−GFP−Hec1_ in IM cells fixed with PFA (113 ± 14 nm; figure 2*c*) or GA (109 ± 14 nm; figure 2*d*) is greater than delta_CenpA-Hec1_ (95 ± 14 nm; figure 2*a*). A similar difference in the distance between Hec1 and CenpA versus CenpA-GFP positions was observed in human cells [10,24] and the difference was attributed to the increased fluorescence between the sister kinetochores due to overexpression of CenpA [24]. In contrast to human cells, where Taxol decreases delta_CenpA-GFP-Hec1_ by approximately 30 nm, the value decreases merely 5–7 nm in IM despite a prominent decrease in the distance between sister kinetochores, which manifests cessation of forces that stretch the centromere (figure 2*c,d*).

Physiological response to 10 µM Taxol appears similar in IM and human RPE1 cells (electronic supplementary material, figure S1). In both cell types, 15 min after addition, density of spindle microtubules increases prominently near the spindle poles and decreases near the equator (electronic supplementary material, figure S1A,B). During prolonged exposure to the drug, both human and IM cells continue to enter mitosis but remain arrested for greater than 20 h and ultimately die without completing cell division (electronic supplementary material, figure S1C, movies S1 and S2). This behaviour indicates that IM cells possess a stringent SAC that is not satisfied despite the absence of a prominent delta decrease in 10 µM Taxol. Thus, our observations are inconsistent with the proposed role of IKT in the control of mitotic progression and/or with the notion that delta is a reliable metrics for IKT.

In contrast to Taxol, delta_CenpA-GFP-Hec1_ decreases by approximately 20 nm after 15 min exposure of IM cells to 3 µM nocodazole (figure 2*c,d*). Under this condition, kinetochores are completely devoid of microtubules (electronic supplementary material, figure S2, 16 kinetochores in two cells). Importantly, inter-kinetochore distances are similar in nocodazole versus Taxol cells (figure 2*c,d*), which indicates that 10 µM Taxol fully supresses microtubule-mediated forces capable of stretching the centromere. Thus, redistribution of molecules within kinetochores that occur upon microtubule attachment makes a stronger contribution to delta than attenuation of microtubule-mediated forces.

### 2.3. ‘Inner' and ‘outer' proteins spatially overlap within IM kinetochores

**Figure 3.**
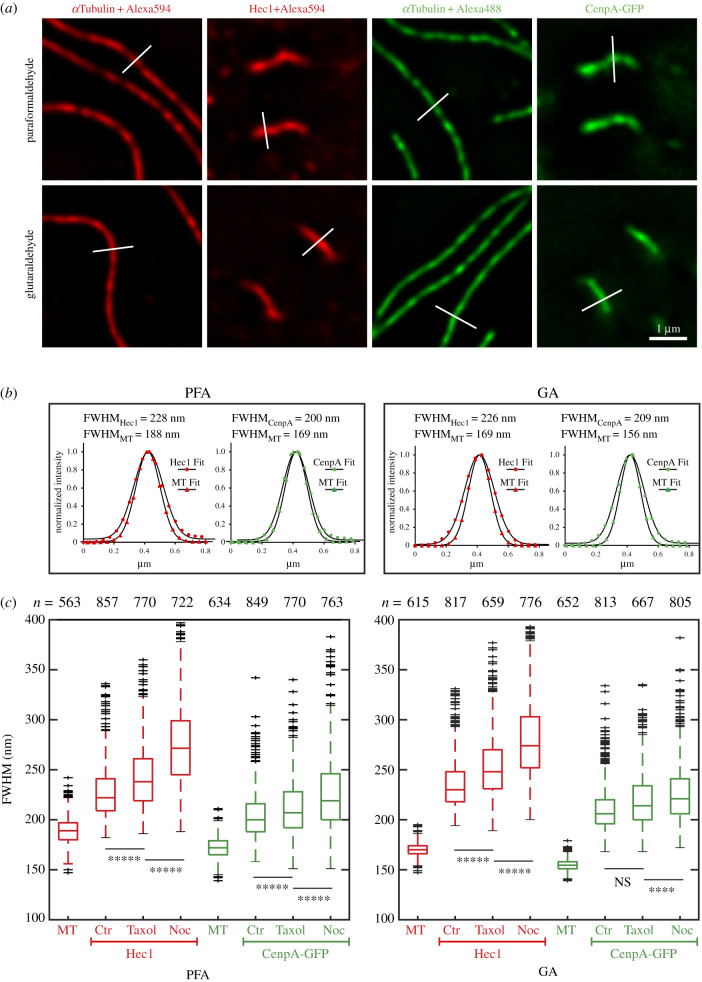
Kinetochore layers in IM cells are wide and they further expand upon Taxol and nocodazole treatments. (*a*) Examples of line-scans (white lines) across individual microtubules or kinetochore plates in cells fixed with paraformaldehyde (PFA) or glutaraldehyde (GA). (*b*) Fluorescence profiles corresponding to line-scans shown in *a*. Markers are pixel intensities, lines are Gaussian fits. Values of the full width at half-maximum (FWHM) are shown for each profile. Note that fluorescence peaks of inner- and outer-kinetochore layers are significantly wider than the peaks of individual microtubules. (*c*) Box plots presenting measurements of FWHM for microtubules, inner- (CenpA-GFP) and outer- (Hec1) kinetochore layers in untreated, Taxol- and nocodazole- treated metaphases after PFA (left) and GA (right) fixation. Note that FWHM of Hec1 layer increases in Taxol- and nocodazole-treated cells. Increase in CenpA-GFP layer is apparent after PFA but not after GA fixation. Student's *t*-test *p*-values are less than 10^−4^ (****), 10^−5^ (*****), or greater than 0.5 (NS).

Morphologically, kinetochores appear as approximately 75 nm trilaminar plates in GA-fixed plastic-embedded EM preparations (figure 1*c,d*). The plates are not detected upon siRNA depletion of Hec1 or when targeting of Hec1 to the kinetochore is abrogated by via depletion of other NDC80 components [27]. These observations, along with immuno-EM that localizes Hec1 to patches on the opposite surfaces of the centromere [26,27], suggest that Hec1 resides primarily within the plate. However, correlative immuno-LM/immuno-EM analyses suggest that Hec1 molecules are spread in the direction of the attached microtubules for greater than 200 nm in human cells [10]. A unique advantage of IM kinetochore is that the width of the spatial domains occupied by various kinetochore proteins can be estimated as the full width at half maximum (FWHM) of the fluorescence peak in line scans orthogonal to the orientation of the plate (figure 3*a,b*). Importantly, due to their large size, plates of IM kinetochores orient vertically (along *Z*-axis) when sister kinetochores are co-planar in *XY* (electronic supplementary material, figures S3 and S4). Co-planar sister kinetochores are reliably identified in 3D LM volumes as both sisters appear as sharp lines in greater than three consecutive optical planes at 200 nm Z-steps (electronic supplementary material, movie S3).

We find that FWHM of the Hec1 distribution within IM kinetochores during metaphase is 233 ± 20 nm after GA fixation and 225 ± 22 nm after PFA fixation (figure 3*c*). These values are significantly larger than FWHM of line scans across individual microtubules (170 ± 7 nm after GA and 189 ± 13 nm after PFA, figure 3*c*), visualized with the same fluorophore and under identical optical conditions. EM firmly establishes that the diameter of a microtubule in GA-fixed cells is approximately 25 nm [28], which implies that FWHM of line scans across a microtubule is determined by the diffraction-limited resolution of the optical system. Thus, the width of Hec1 distribution, which exceeds FWHM of diffraction-limited profile by greater than 60 nm, reflects the true physical width of the layer occupied by Hec1. Importantly, FWHM values are significantly more variable among Hec1 than among microtubule profiles (figure 3*c*). This increased variability supports the notion that the width of microtubule profiles is determined by the optics while the Hec1 profiles reflect natural fluctuations in the organization of the kinetochore plate. We also note that the width of microtubule profiles in PFA-fixed samples is greater than after GA fixation. This increase likely reflects disintegration of microtubule structure that results in spatial redistribution of tubulin. Indeed, microtubules are not structurally detectable in EM on PFA-fixed samples [28,29].

Measurements in metaphase cells treated with Taxol demonstrate that abrogation of centromere tension increases FWHM of Hec1 to 251 ± 27 nm after GA and 241 ± 30 nm after PFA fixation. Complete depolymerization of microtubules with a high concentration of nocodazole (electronic supplementary material, figure S2) results in a more prominent increase of Hec1 FWHM to 278 ± 35 nm (GA) and 273 ± 39 nm (PFA) (figure 3*c*).

CenpA-GFP also localizes within a layer of measurable width. Because LM resolution depends on the wavelength, FWHM is less for a diffraction-limited peak formed by a green versus red fluorophore. On the microscope used in this study, FWHM of microtubules visualized with a green fluorophore (GFP or Alexa488) is 155 ± 6 nm after GA and 172 ± 11 nm after PFA fixation (figure 3*c*). CenpA-GFP peaks are significantly wider, measuring 208 ± 18 nm after GA fixation and 202 ± 19 nm after PFA. The width of CenpA-GFP layer increases slightly to 216 ± 24 nm (GA) and 210 ± 26 nm (PFA) in Taxol-treated cells, and further increases to 224 ± 24 nm (GA) and 222 ± 30 nm (PFA) when microtubules are completely depolymerized with nocodazole (figure 3*c*).

FWHM measurements demonstrate that both inner (CenpA) and outer (Hec1) kinetochore components reside within layers whose widths are significantly larger than the diffraction limit of resolution and therefore the widths of these layers are measurable in LM. Furthermore, CenpA and Hec1 layers are approximately twofold larger than the distance between the centres of the layers occupied by these proteins (i.e. Delta). These dimensions imply that approximately half of CenpA and Hec1 molecules are spatially intermixed within the same compartment, which in turn means that delta does not accurately reflect the average distance between molecules within IM kinetochores.

### 2.4. ‘Inner' and ‘outer' proteins spatially overlap within kinetochores in human cells

Our observation that the width of the layers formed by inner and outer proteins within compound kinetochores of IM exceeds 200 nm raises a question of whether a similar architecture exists in human kinetochores. The principal assumption in interpretation of kSHREC measurements is that kinetochore proteins form negligibly thin layers within an approximately 300 nm-long plate [7]. A corollary of this assumption is that FWHM of the kinetochore spots in LM should reflect the length of the plate in one direction and be diffraction limited in the orthogonal direction.

**Figure 4.**
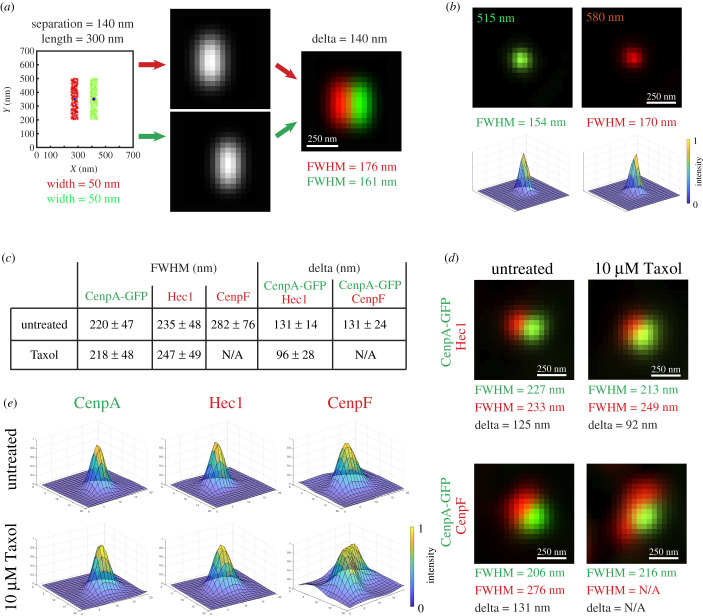
Kinetochore layers in human kinetochores are wide and spatially overlap. (*a*) Layout of computational model used to predict LM appearance of human kinetochores. Randomized distribution of molecules within a specified 3D shape is convolved with wavelength-specific Gaussian point-spread function (PSF). Convolved volumes are then downscaled to the voxel size typical in conventional LM. Predicted LM appearance shown for kinetochores shaped as 300 × 250 nm rectangular prism with 50 nm-wide layers separated by 140 nm. Delta and FWHM values are measured in pseudo-LM volume constructed at 40 nm *XY* pixels and 200 nm Z-steps. (*b*) LM images and intensity profiles of a 100 nm multicolour bead shown in green (515 nm) and red (580 nm) colours. FWHM is reported for the longer axis in *XY* plane. (*c*) Delta and FWHM values measured in RPE1 cells prepared under the same conditions as IM cells. More than 100 centromeres from greater than 3 cells for each experimental condition. (*d–e*) LM images (*d*) and intensity profiles (*e*) of a human kinetochore with typical values of delta and FWHM. Note that both inner (CenpA-GFP) and outer (Hec1, CenpF) domains are significantly larger than a diffraction-limited spot. Delta and FWHM for CenpF spots cannot be measured in Taxol as their complex shape is not Gaussian.

To explore whether fluorescently labelled kinetochores in human cells resemble the shape and dimensions assumed in kSHREC analyses, we constructed a computational simulation in which the inner and outer kinetochore layers are modelled as a specified number of ‘molecules' (points) randomly distributed within a 3D volume of specified shape. This distribution of molecules is convolved (blurred) with a 3D Gaussian filter which mimics the point spread function (PSF) of a light microscope. The blurred image is then scaled down to match dimensions of voxels in a typical LM volume recorded on a CCD camera at specified *Z*-steps. Fitting a Gaussian function to these simulated 3D images of kinetochores is then used to determine coordinates and FWHM of the peaks (figure 4*a*).

To establish PSF parameters, we recorded 3D volumes of multi-colour 100 nm fluorescent beads under the same optical conditions as kinetochores (figure 4*b*). Measurements of the beads yielded 155 ± 27 nm (*n* = 245) FWHM in the green and 172 ± 31 nm (*n* = 125) in the red channels. These FWHM values are indistinguishable from FWHM of individual microtubules determined by orthogonal line-scans in GA-fixed samples (figure 3*c*) as expected for measurements of diffraction-limited objects on the same microscope. With the experimentally measured PSF parameters, the model predicts that kinetochores comprising inner and outer layers with diffraction-limited widths and separated by delta previously observed in RPE1 cells [10] should appear as drastically elongated fluorescent spots with minimal overlap between the layers (figure 4*a*). In contrast to this prediction, spots formed by various kinetochore proteins in human cells are significantly larger than images of sub-resolvable beads, with mean FWHM values exceeding that of the PSF by approximately 65 nm for CenpA-GFP or Hec1 and approximately 112 nm for more peripheral components such as CenpF (figure 4*c*). Furthermore, in contrast to the uniform appearance of sub-resolvable beads, kinetochore spots display variable shapes; however, most kinetochores do not resemble elongated fluorescent spots predicted by the model (electronic supplementary material, figure S5, movies S4–S6). CenpA-GFP, Hec1 and CenpF spots of kinetochores that display average values of delta and FWHM appear sufficiently round (figure 4*d*). In Taxol-treated cells, the shapes of Hec1 and CenpF spots become irregular (electronic supplementary material, figure S6, movies S4–S6) to the extent that CenpF spots cannot be fitted with a Gaussian function at a reasonable residual error (<10%). Although precise characterization of the observed changes in shape of human kinetochores in response to Taxol is impossible due to their variable orientation and anisotropic resolution of LM, Hec1 and CenpF often appear as crescents with variable curvature (figure 4*e*; electronic supplementary material, figure S6).

FWHM of human kinetochore spots indicate that inner and outer kinetochore proteins spatially overlap, akin to the 50% overlap between CenpA-GFP and Hec1 layers observed in IM cells (figure 3). The large width of overlapping layers as well as variability in the shape of the kinetochore spots may profoundly affect interpretation of kSHREC measurements in human cells with undiscernible orientation of kinetochores.

### 2.5. Taxol treatment increases the length and curvature of kinetochore plates in human cells

**Figure 5.**
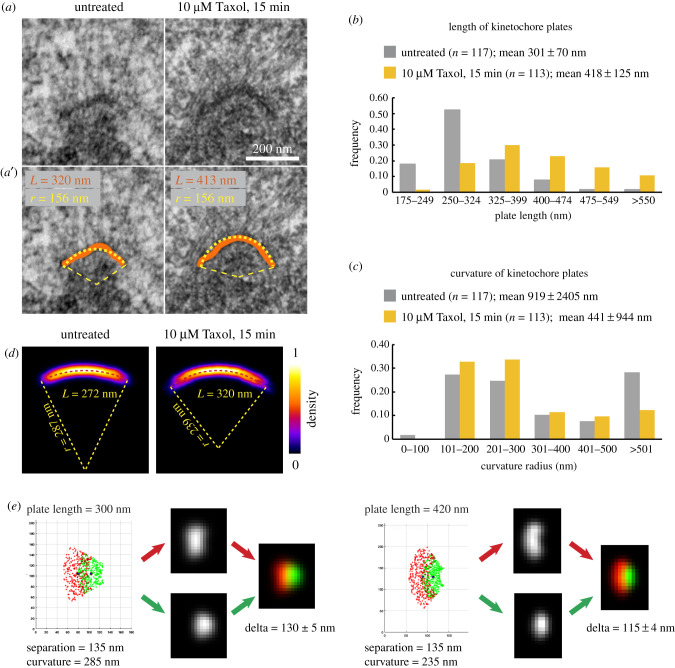
Geometry of the kinetochore plate and its effects on delta in human cells. (*a*-*a*′) 70-nm EM sections containing kinetochore plates in untreated (left) versus Taxol-treated RPE1 cells. Orange contours tracing the 75 nm trilaminar plates and fit lines of a circular arc (yellow) are shown in (*a*′). (*b*) Frequency histogram for plates with various lengths in untreated and Taxol-treated cells. *n* is number of kinetochores from greater than 3 cells reconstructed for each condition. (*c*) Frequency histogram for plates with various curvature determined by fitting individual plates with circular arcs. Note that 70% of kinetochores display curvature radius less than 300 nm. (*d*) Results of individual plate averaging in untreated (left) versus Taxol-treated cells (right). Dashed lines indicate perfect circular segments with the shown radii. (*e*) Predicted change in the LM appearance and delta due to increased length and curvature of the plate.

Canonical interpretation of kSHREC as a metric for distance separating layered proteins stems from the thin-plate morphology of kinetochores in conventional EM. Two lines of evidence identify the 75 nm trilaminar plate as the primary locale for the microtubule-binding kinetochore components: (1) the great majority of end-on attached microtubules terminate within the plate [30]; and (2) depletion of Hec1 and/or other Ndc80 components renders the plate morphologically indistinct [27]. Previous computational analyses suggest that for inflexible disc-like plates delta accurately reflects the average distance between molecules even if the layers are slanted or jagged [7]. However, for layers shaped as crescents, the value of delta is smaller than the distance between the layers. This difference increases for longer crescents and higher curvatures. Systematic analysis of the kinetochore plate length and curvature in EM preparations has not been reported, which prompted us to assess geometry of the plates in untreated (electronic supplementary material, figure S7A) versus Taxol-treated RPE1 cells via serial 70 nm plastic sections (electronic supplementary material, figure S7B). The length was measured by tracing the plate in the medial section and the curvature was estimated by fitting traced contour of the plate with a circular segment (figure 5*a,a′*).

Consistent with the variability observed in LM (electronic supplementary material, figures S5 and S6), both the length and shape of the kinetochore plate are highly variable in EM (electronic supplementary material, figure S8). The mean value of plate length is 301 ± 70 nm (117 kinetochores from 3 cells) during metaphase and it increases to 418 ± 125 (113 kinetochores from 4 cells) when metaphase cells are treated with Taxol for 15 min (figure 5*b*). Importantly, Taxol treatment results in the appearance of extremely long kinetochores (up to 1159 nm) with curved plates (electronic supplementary material, figure S8). Undulations, protrusions and cavitation are prominent in most plates (electronic supplementary material, figure S8), and these irregularities complicate assessment of the typical plate curvature. While longer plates with visually apparent bending fit well with a circular arc (figure 5*a*), shorter and relatively straight plates yield a wide range of curvature values. As a result, the distribution of values is not normal, which confounds comparisons of the means (figure 5*c*). However, this approach demonstrates that half of kinetochore plates display curvatures < 275 nm in untreated RPE1 cells. The median curvature decreases to 235 nm in cells exposed to Taxol for 15 min. To circumvent high variability of curvatures measured on individual plates, we average numerous kinetochore plates after rotationally aligning the base of the kinetochore (line connecting opposite termini of the plate) and translationally superimposing the outmost central point of the plate. This approach suggests that the overall shape of a typical plate resembles a circular arc with curvature radii 287 nm in the untreated and 239 nm in Taxol-treated cells (figure 5*d*).

EM analyses suggest that a typical kinetochore plate is shaped as an outward-oriented concave segment on the centromere surface. Taxol significantly increases the plate length and its curvature, which would decrease the value of delta even if the average distance between the outer and inner kinetochore domains remain constant. High variability in the shape and dimensions among human kinetochores complicates assessment of the exact contribution from architectural changes towards delta. However, for an idealized uniform population of kinetochores, observed changes in the plate geometry would decrease delta by approximately 15 nm (figure 5*e*), which accounts for approximately 50% of the decrease observed in this study (figure 4*c*) as well as in previous reports [7,10].

## 3. Discussion

Pioneering time-lapse recordings of live cells with kinetochores labelled in two different colours revealed that the distance between fluorescent spots corresponding to the microtubule-binding domain and the base of kinetochore was not constant. Specifically, the spots appeared to be closer together when microtubule dynamics were suppressed with Taxol, a treatment known to reduce the force that stretches centromeres of bioriented chromosomes [8,31]. These observations gave birth to the concept of IKT [9]. Subsequent pairwise measurements of distances between the spots formed by various kinetochore proteins (i.e. delta) laid the foundation of a linear map that ascribed various proteins to specific layers within the kinetochore and interpreted delta as the distance that separates these layers [7]. Essential for this interpretation of delta was the assumption that kinetochores are shaped as flat thin discs and this architecture remains constant. Here, we tested this assumption and found that human kinetochores are not flat and that curvature as well as the length of the plate increase in Taxol-treated cells. These architectural changes, consistent with the earlier EM descriptions of kinetochore plates (reviewed in [32]), imply that delta underestimates the distance between the outer versus inner layers and the magnitude of underestimation is different under various conditions. Thus, delta is not a quantitative metric for IKT in human cells.

Consistent with the notion that large-scale architectural reorganizations contribute significantly towards the value of delta in human cells, we observe that delta remains virtually constant in Taxol-treated IM cells. Increased dimensions of the plate make the shape and orientation of IM kinetochore discernible in LM, which enables measurements of delta that are largely independent from malleable reorganization of kinetochore architecture and thus primarily reflect true changes in IKT. Importantly, delta in IM decreases significantly in the absence of microtubule attachments, which indicates that the measurements are sufficiently sensitive to detect redistribution of proteins within the kinetochore plate. Thus, the absence of prominent delta changes in IM upon suppression of microtubule forces by Taxol implies one of the two possibilities. First, mechanic properties of IM kinetochores may fundamentally differ from those in humans. Second, the large decrease in Delta observed in human cells to a large degree reflects changes in overall shape/size of the kinetochore and not the distance between the layers of molecules. Similar physiological response of IM and human cells to Taxol, similar molecular composition of kinetochores [21], similar morphology of kinetochore plates in both species (figure 1), and similar values of delta in untreated cells (cf. figure 2 and [7]) render the first possibility unlikely. Therefore, decrease in delta values observed in human cells appears reflect both larger scale non-elastic changes within the malleable outer plate and the distance between the layers of molecules (i.e. IKT). Computational modelling suggests that contributions from shape changes are as large as approximately 50% (figure 5*e*). This interpretation gains additional support from observations of different delta values in sister kinetochores [10,31,33]. It is impossible for linearly connected elastic elements to simultaneously experience different levels of tension. By contrast, changes in the shape of kinetochore plate are expected to be independent for sister kinetochores and prominent differences in the shape of sister kinetochores are directly observed in EM (electronic supplementary material, figure S7).

In recent years, kSHREC analyses have led to contradictory conclusions regarding the nature of IKT as well as its role in mitotic progression. Some studies attribute 100% of changes in delta to changes in the distance between various kinetochore proteins or conformational changes within a specific molecule [7]. Others conclude that delta in part [10] or exclusively [12] reflects swivelling (angular reorientation) of kinetochores on the centromere instead of reporting correct intrakinetochore distances. These discrepancies are exaggerated by large differences in absolute values of delta for the same pair of proteins that vary as much as 40% in different reports [6,7,10,12]. In part, this lack of consensus may be due to different approaches to delta measurements employed in different laboratories and there is a thoughtful debate on the conditions necessary for accurate localization of fluorescent spots in cells [6,13]. However, a larger issue is that interpretation of delta requires a strict model of layered kinetochore organization, which lacks solid experimental support. FWHM of Hec1 and CenpA peaks in IM cells demonstrate a significant (50% or greater) spatial overlap of ‘inner’ and ‘outer' proteins, which is inconsistent with the notion of negligibly thin layers that underlies kSHREC. A similar overlap appears to exist in human cells, although FWHM measurements are less reliable in these cells due to complexity and variability in the shape of kinetochore spots. However, it is clear that human kinetochores are significantly larger than the resolution limit of conventional LM and this urges caution in literal interpretation of changes in delta as a metric for IKT.

## 4. Material and methods

### 4.1. Cell culture and drug treatments

Immortalized hTERT-RPE1 constitutively expressing CenpA-GFP and Centrin-1-GFP [34] and hTERT-Indian muntjac cells ([21], kind gift of Dr Helder Maiato, Institute of Molecular and CellularBiology, Porto, Portugal) were cultured in DMEM (Gibco, Life Technologies) supplemented with 10% FBS (Sigma-Aldrich F0926) at 37°C and 5% CO_2_. Taxol (Paclitaxel T7402–5MG; Sigma-Aldrich) and nocodazole (Calbiochem, #487928) were added approximately 15 min prior to fixation to final concentrations of 10 μM and 3 μM, respectively.

### 4.2. Time-lapse recordings

Cells were seeded in 12.5 cm^2^ tissue culture flasks and grown in DMEM (Gibco, Life Technologies) supplemented with 10% FBS (Sigma-Aldrich F0926) and 25 mM HEPES (Gibco by Life Technologies REF 15630–080) at 37°C and 5% CO_2_ for 24 h. Taxol was added in conditioned culture media to 5 μM final concentration, 30 min prior to filming. Cells were imaged on Nikon TS100 microscopes equipped with a 10× objective lens (Nikon) at 37°C for 48–72 h. Phase-contrast or differential interference contrast images were captured at 2 min intervals on a Monochrome Spot IR camera. The microscope and light source were controlled by Spot 5.3 Advanced software (Diagnostic Instruments, Inc.).

### 4.3. Immunofluorescence

Cells were concurrently permeabilized and fixed in PEM buffer (100 mM Pipes, pH 6.9, 2.5 mM EGTA, and 5 mM MgCl2, pH 6.9) supplemented with 1% Triton X-100 and 1% glutaraldehyde (G5882; Sigma-Aldrich) or 1% Triton X-100 and 3.2% paraformaldehyde (EM grade; EMS) for 10 min. Microtubules were visualized with DM1*α* monoclonal anti-α-tubulin antibody at 1 : 100 (T9026; Sigma-Aldrich) followed by a secondary antibody conjugated with Alexa Fluor 594 (A-11032; Thermo Fisher Scientific) or Alexa Fluor 488 (A-11029, Thermo Fisher Scientific). Outer kinetochore domain was stained with a monoclonal 9G3/Hec1 antibody (Abcam ab3613) at 1 : 1000 or a rabbit polyclonal antibody against a C-terminal of CenpF (NB500–101; Novus Biological at 1 : 300 followed by an appropriate secondary antibody conjugated with Alexa Fluor 594 (Thermo Fisher Scientific). In the experiments that involved immunostaining for endogenous CenpA, the cells were fixed in paraformaldehyde and postfixed in 100% methanol at –18°C for 15 min. Endogenous CenpA was visualized with a mouse monoclonal antibody (3–19, Abcam; ab13939) at 1 : 200. Although both 3–19 (CENP-A) and 9G3 (Ndc80/Hec1) antibodies are mouse monoclonal, their isotypes are distinctly different, which allows highly specific co-staining. 3–19 antibody was detected with IgG1 (*γ*1)-specific secondary antibody conjugated to Alexa Fluor 488 (Thermo Fisher Scientific, A-21121;), and 9G3 was detected with IgG2a (*γ*2a) specific secondary antibody conjugated to Alexa Fluor 594 (Thermo Fisher Scientific, A-21135;), both at 1 : 100 dilution.

Immunostained cells were embedded in non-solidifying media containing 90% glycerol, 10% 1 M Tris–Cl, pH 8.5, and 1 mg ml^−1^ PPDA. Wide-field fluorescence images were obtained on a Nikon TE2000E2 microscope equipped with 100× 1.49 NA PlanApo TIRF lens and LED illuminator (CoolLED PE 4000). Images were captured with an Andor Zyla 4.2 camera at 43 nm *X*-*Y* pixels and 200 nm *Z* steps. The system was controlled by Nikon NIS-Elements Advanced Research software (Nikon instruments). All images were deconvolved with SoftWorRx 5.0 deconvolution software (Applied Precision), and objective lens-specific in house-recorded point-spread functions. Deconvolution method was set to ‘Conservative', noise level to ‘Medium' for kinetochores and ‘Low' for 100 nm beads, and the process ran for 10 iterations.

### 4.4. Serial section electron microscopy

IM and RPE1 cells were fixed with 2.5% glutaraldehyde (G5882; Sigma-Aldrich) in PBS, pH7.4 for 30 min, rinsed with PBS (3 × 5 min), and post-fixed with 2% OsO_4_ in dH_2_O for 60 min at 4°C. The coverslips were then rinsed in dH_2_O, treated with 0.25% tannic acid for 20 min, and stained with 2% uranyl acetate for 60 min. Dehydration was achieved by a series of ethanol solutions (30–50–70–80–96%, 10 min in each solution) followed by acetone (10 min). After dehydration, cells were embedded in Epon 812 and cured for 48 h at 60°C. Serial 70 nm thickness sections were cut with a diamond knife (DiATOME) on a Leica Ultracut UCT ultramicrotome and stained with lead citrate. Images were obtained on a JEOL 1400 microscope operated at 80 kV using a side-mounted 4.0 Megapixel XR401 sCMOS AMT camera (Advanced Microscopy Techniques Corp). Higher-magnification images (40 K) were collected for individual kinetochores. These high-magnification images were subsequently used to trace kinetochores plates. 3-D volumes occupied by the kinetochores were visualized as isosurface models in Amira 5.3.3 (Visage Imaging).

### 4.5. Measurement of interkinetochore distances, delta, and full width at half maximum in IM cells

Intensity profiles of selected centromeres (CenpA-GFP) with co-planar sister kinetochores (Ndc80/Hec1) were obtained for single-pixel lines in ImageJ. Precise position and FWHM of the two Gaussian peaks in each of these profiles were obtained via the Peakfit MATLAB function developed by Dr O'Haver (https://www.mathworks.com/matlabcentral/fileexchange/23611-peakfit-m). Profiles with fit errors larger than 3% were discarded (less than 3% of all obtained profiles).

Interkinetochore distance (IKD) was calculated by subtracting the distance between the centres of CENP-A or Hec1 peaks. Delta values were calculated by subtracting the distance between the centres of CENP-A peaks from the distance between the centres of Ndc80/Hec1 peaks and dividing the result by 2. In this approach, potential errors due to chromatic aberration are automatically negated by the opposite orientation of sister kinetochores. However, the same (average) value of delta is assigned to both sister kinetochores.

### 4.6. Measurement of interkinetochore distances, delta, and full width at half maximum in RPE1 cells

Precise positions and dimensions of fluorescent spots in RPE1 cells were obtained with the delta_tool Matlab function described in reference [10] and available at http://www.jcb.org/cgi/content/full/jcb.201412139/DC1. This function determines *x*-*y* coordinate of the kinetochores centre of mass via nonlinear fitting (lsqcurvefit Matlab function) in projections of segmented volumes of kinetochores with a 2D Gaussian function. Z coordinates are calculated separately in *x*-*z* projections. Chromatic aberration errors are suppressed by shifting one colour channel so that the global centres of mass for the red and green channels coincide. Delta is then calculated directly as the distance between the red and green centroids within the same kinetochore.

### 4.7. Measurement of the outer plate length and curvature

Length of the kinetochore outer plate was determined by tracing the contours of the plate with the Freehand line tool in ImageJ (National Institutes of Health). Contours of the plate were fitted with a circular arc function via Matlab function developed by Dr Nikolai Chernov, University of Alabama, Birmingham, AL (https://www.mathworks.com/matlabcentral/fileexchange/22643-circle-fit-pratt-method).

For plate averaging, individual plates traced at 1.88 nm pixel^−1^ were rotated to place both termini of the plate on a horizontal line. Rotated images were translated to align outmost central point of each contour. Average-intensity projection was then calculated and displayed at single precision (32-bit). The single-pixel chords of the averaged plates were fitted with a circular segment to determine curvature radii as described in the previous paragraph.

### 4.8. Computational simulation for evaluation LM appearance of kinetochores with various architecture

The simulation was made using a Matlab code, and the following parameters are considered. Kinetochores are modelled as two plates, each consisting of fixed number of molecules uniformly distributed randomly within a 3D volume of specified dimensions and position relative to the other plate with the possibility of overlap. The volume is rectangular except for curvature in either the *XY* or *XZ* planes with assumed rotational symmetry with respect to both the *Z* and *Y* axes. Two PSFs for ‘green' and ‘red' colours are constructed by convolving a single point with 3D Gaussian filter (implemented via fspecial3 function introduced in 2018b version of Matlab). Widths of the PSFs were specified independently in the *XY* plane and along the Z direction. Convolved volumes were downsampled to the *XY* pixel size and *Z* steps expected in LM by integrating intensity constrained within the part of convolved volume that corresponds to the specified LM voxel. Relative position of the downsampled LM array with respect to the full-resolution convolved volume was randomized within 1 LM pixel to mimic random positions of kinetochores with respect to CCD pixels. Pseudo-experimentals measurements of delta and FWHM were conducted as in real experiments but within the simulated volume at LM resolution.

### 4.9. Preparation of illustrations

Contrast and brightness of the final LM images were linearly adjusted in Photoshop 2020 and the figures assembled in Illustrator 2020 (Adobe). Graphs were prepared in Matlab or Excel and imported into Illustrator as PDFs.

## Supplementary Material

supplemental_figures.docx

## Supplementary Material

movie_legends.docx

## Supplementary Material

movie_1.mov

## Supplementary Material

movie_2.mov

## Supplementary Material

movie_3.mov

## Supplementary Material

movie_4.mov

## Supplementary Material

movie_5.mov

## Supplementary Material

movie_6.mov

## Data accessibility

This article has no additional data. The authors adhere to the NIH Grants Policy and Sharing of Unique Research Resources. All research resources, including protocols, constructs, cell lines, chemical probes and Matlab codes, are available upon request from the corresponding author.
