# Supplementary material for: Effects of malleable kinetochore morphology on measurements of intrakinetochore tension
Source: Open Biol. 2020 Jul 8;10(7):200101. doi: 10.1098/rsob.200101 (PMC7571466; doi:10.1098/rsob.200101)
Supplement: supplemental_figures.docx [file rsob200101supp1.docx]

**Supplementary material**

**Open Biology, DOI:10.1098/rsob.2016 XXXX**

**Effects of malleable kinetochore morphology on measurements of intrakinetochore tension**

Fioranna Renda, Valentin Magidson, Irina Tikhonenko, Rebecca Fisher, Christopher Miles, Alex Mogilner, and Alexey Khodjakov.

**Figure S1. Effects of Taxol in IM and human cells.** **(A - B)** Similar changes in mitotic spindle architecture in human RPE1 (A) and IM (B) cells. Approximately 15 min after addition of Taxol (10-μM final concentration), already assembled mitotic spindles remain bipolar with congressed chromosomes in both human and IM cells. Microtubules density decreases near chromosomes and increases near spindle poles (A) Maximum-intensity projection (MIP) of an untreated (top) and Taxol-treated (bottom) RPE1 metaphases. (B) MIP of an untreated (top) and Taxol-treated (bottom) IM metaphases. Kinetochores are labelled via expression of CenpA-GFP (green); microtubules are immunostained with a monoclonal antibody (DM1A, Sigma) against α-tubulin (red); chromosomes are stained with DNA dye Hoechst 33342 (blue). RPE1 cells additionally express Centrin1-GFP that marks positions of centrioles/spindle poles (arrowheads in A). **(C)** Stringent mitotic arrest in RPE1 (top) and IM (bottom) in the presence of 5‑μM Taxol. In both cell types mitosis is arrested for >20 hrs and most cells subsequently die. Selected movie frames from a 48-hr long time-lapse recording at 2-min intervals (see Electronic supplementary material, movies 1 and 2 for full recordings).

**Figure S2. Morphology of the kinetochore plates in nocodazole-treated Indian muntjac prometaphase.** 90-nm serial EM sections from a full series through the centromere region of the chromosome. IM cells treated with 3-μM nocodazole for 15 min exhibited unattached kinetochores. Notice that all microtubules were depolymerized after this treatment. Overall morphology of the plates (orange arrows) resembles that in untreated metaphase (figure S3).

**Figure S3. Morphology of the kinetochore plates in untreated Indian muntjac metaphase.** 70-nm serial EM sections from a full series through the centromere region of the chromosome. Notice the length of plate (orange arrows) and a large number of microtubules attached to the kinetochores. 3D reconstruction of the plate (bottom, right) demonstrates lack of bending in the vertical plane and that plates of co-planar sister kinetochores orient approximately orthogonal to the sectioning plane.

**Figure S4. Morphology of the kinetochore plates in Taxol-treated Indian muntjac metaphase**. Similar to Figure S3 but this cell was exposed 10-μM Taxol for 15 min. Notice that overall morphology of the plates resembles that in untreated metaphase (figure S3) although the number of attached microtubules decreases.

**Figure S5.** **Variability in size and shape of fluorescent spots formed by kinetochore proteins in untreated RPE1 cells.** CenpA-GFP **(A**), Hec1 **(B)**, and CenpF **(C)** spots randomly selected from untreated metaphase cells. Full 3-D volumes of these cells are shown in movies 4-6 (see Electronic supplementary material). Numbers correspond to arbitrary indices assigned to individual chromosomes. ‘a’ and ‘b’ denote sister kinetochores oriented towards opposite spindle poles. Orientations of metaphase plate and spindle axis are shown in the first square of each panel. Notice that the spots are considerably larger than diffraction-limited spots formed by 100-nm fluorescent beads recorded under identical optical conditions (second square of each panel). All images are shown at individually normalized intensities to enable visual comparison.

**Figure S6. Variability in size and shape of fluorescent spots formed by kinetochore proteins in Taxol-treated RPE1 cells.** Similar to Figure S5 but the cells were exposed to 10-μM Taxol for 15 min. Notice prominent increase in size of the spots formed by outer kinetochore proteins Hec1 (B) and CenpF (C). Complexity and variability to the shapes displayed by CenpF prevents reliable calculation of the centroid via Gaussian fitting. Apparent size and shape of CenpA-GFP spots in Taxol-treated cells (A) is similar to that in untreated cells (see figure S5). All images are shown at individually normalized intensities to enable visual comparison.

**Figure S7.** **Examples of serial-section reconstruction of sister kinetochore from untreated and Taxol-treated metaphase RPE1 cells.** **(A)** Selected serial 70-nm EM sections through the centromere. Numbers indicate relative depth of each section within the volume. Arrows denote the outer electron dense layers of the plates that were traced and segmented to construct a 3-D surface-rendered model shown in (A’). **(B-B’)** As in panels A-A’ but the cell was exposed to 10-μM Taxol for 15 min. Notice increased size of the plates.

**Figure S8. Variability in the shape of kinetochore plates in RPE1 cells. (A)** Contours of 117 kinetochore plates from 3 untreated metaphase cells. **(B)** Contours of 113 kinetochore plates from 4 metaphase cells exposed to 10-μM Taxol for 15 min. Each contour was traced in a 70-nm section near the middle of the plate. All plates shown in these panels were used to calculate averages shown in Figure 5D.
