# Supplementary material for: Effects of malleable kinetochore morphology on measurements of intrakinetochore tension
Source: Open Biol. 2020 Jul 8;10(7):200101. doi: 10.1098/rsob.200101 (PMC7571466; doi:10.1098/rsob.200101)
Supplement: movie_legends.docx [file rsob200101supp2.docx]

**Electronic supplementary material**

**Open Biology, DOI:10.1098/rsob.2016 XXXX**

**Effects of malleable kinetochore morphology on measurements of intrakinetochore tension**

Fioranna Renda, Valentin Magidson, Irina Tikhonenko, Rebecca Fisher, Christopher Miles, Alex Mogilner, and Alexey Khodjakov.

## Movie legends

**Movie 1. Effects of Taxol on RPE1 cells.** Cells that enter mitosis (manifested by the characteristic round morphology) in the presence of 5-μM Taxol, remain arrested and eventually die. Phase-contrast microscopy. Time stamp in hours: minutes. Selected time points from this recording are shown in Figure S1C (see Electronic supplementary material).

**Movie 2. Effects of Taxol on IM cells.** Taxol treatment and filming conditions identical to Movie 1. Selected time points from this recording are shown in Figure S1C (see Electronic supplementary material).

**Movie 3. Typical orientation of kinetochores in IM cells.** 200-nm steps through 3D volumes of an untreated (left) and Taxol-treated (10-µM, 15-min) metaphase cells. Each kinetochore is labelled in green (CenpA-GFP) and red (Hec1 visualized with Alexa594). Chromosomes are stained with Hoechst 33342 (blue). Scale bars apply to X–Y planes; depth of each plane is denoted in the top-left corners of each frame. Notice that co-planar sister kinetochores appear as approximately anti-parallel sharp lines in greater than three consecutive optical planes. Middle planes through such co-planar kinetochores were selected for Delta and FWHM measurements. Selected kinetochores from these cells are shown in figures 1B-B’ and 2B-B’.

**Movie 4. Distribution and appearance of kinetochores in RPE1 cell.** 200-nm steps through 3D volumes of an untreated (left) and Taxol-treated (10-µM, 15-min) metaphase cells. Numbers denote centromeres with sister kinetochores indexed as ‘a’ and ‘b’. Each kinetochore is labelled in green (CenpA-GFP) and red (Hec1 visualized with Alexa594). Centrosomes (a and b) are labelled with Centrin1-GFP. Chromosomes are stained with Hoechst 33342 (blue). Scale bars apply to X–Y planes; depth of each plane is denoted in the top-left corners of each frame. CenpA-GFP spots of randomly selected kinetochores from these cells are shown in Figures S5A and S6A (see Electronic supplementary material).

**Movie 5. Distribution and appearance of kinetochores in RPE1 cell.** Similar to Movie 4. Hec1 spots of randomly selected kinetochores from these cells are shown in Figures S5B and S6B (see Electronic supplementary material).

**Movie 6. Distribution and appearance of kinetochores in RPE1 cell.** Similar to Movie 4 except the outer kinetochores are visualized via CenpF immunostaining. CenpF spots of randomly selected kinetochores from these cells are shown in Figures S5C and S6C (see Electronic supplementary material).
